# Supplementary material for: An 85-year record of glacier change and refined projections for Kennicott and Root Glaciers, Alaska
Source: Nat Commun. 2025 Aug 22;16:7835. doi: 10.1038/s41467-025-62962-w (PMC12373804; doi:10.1038/s41467-025-62962-w)
Supplement: Supplementary file 1 — Supplementary Information [file 41467_2025_62962_MOESM1_ESM.pdf]

Supplementary Material

An 85-year record of glacier change and refined projections for Kennicott and Root Glaciers, Alaska

Albin Wells*, Brandon S. Tober, Sarah F. Child, David R. Rounce, Michael G. Loso, Chad P. Hults, Martin Truffer, John W. Holt, and Michael S. Christoffersen

*** Correspondence:** Albin Wells: awwells@cmu.edu

TABLE OF CONTENTS

1. Supplementary Notes

- **Supplementary Note 1:** Glacier-wide mass loss *(Page 2)*
- **Supplementary Note 2:** Previous studies on Kennicott and Root Glaciers *(Page 2)*
- **Supplementary Note 3:** Historical image processing settings *(Page 2)*
- **Supplementary Note 4:** Historical glacier-wide mass balance *(Page 3)*

2. Supplementary Tables

- **Supplementary Table 1:** Historical image processing primary statistics *(Page 3)*
- **Supplementary Table 2:** Radar-derived ice thickness crossover disagreement *(Page 4)*
- **Supplementary Table 3:** Historical image processing primary statistics *(Page 5)*
- **Supplementary Table 4:** Radar-derived ice thickness crossover disagreement *(Page 5)*

3. Supplementary Figures

- **Supplementary Fig. 1:** Radar-derived bed elevation and ice thickness *(Page 6)*
- **Supplementary Fig. 2:** Driving stress maps *(Page 7)*
- **Supplementary Fig. 3:** 1957 and 1962 orthophotos and derived velocity *(Page 7)*
- **Supplementary Fig. 4:** 1957 and 2023 glacier outline and debris cover extent *(Page 8)*
- **Supplementary Fig. 5:** Modeled mass change from 1940-2100 *(Page 8)*
- **Supplementary Fig. 6:** Example fiducial marks in historical imagery *(Page 9)*
- **Supplementary Fig. 7:** Schematic of historical DEM processing workflow *(Page 9)*
- **Supplementary Fig. 8:** Aerial photograph and ground-control point locations *(Page 10)*
- **Supplementary Fig. 9:** Hole-filling techniques for ASTER DEM *(Page 10)*
- **Supplementary Fig. 10:** DEM co-registration accuracy and results *(Page 11)*
- **Supplementary Fig. 11:** Elevation differences between the 1957 USGS topographic DEM and the 1957 DEM produced in this study *(Page 12)*
- **Supplementary Fig. 12:** Historical and modern model calibration parameters *(Page 13)*
- **Supplementary Fig. 13:** Observed and modeled binned elevation change rates *(Page 13)*
- **Supplementary Fig. 14:** Modeled equilibrium-line altitude from 1940-2022 *(Page 14)*

4. Supplementary Material References

1 SUPPLEMENTARY TEXT

Supplementary Note 1: Glacier-wide mass loss

To assess the representation of glacier-wide mass changes, we used glacier-wide elevation change maps from 2000-2019[1]. The total mass change was calculated in the ablation areas and glacier-wide for Kennicott and Root Glaciers (Fig. 2g) using a density of 900 kg m^{-3} and 850 kg m^{-3} for the ablation area and the full glacier, respectively. The areal coverage presented in the study represents 95.1% of total mass loss on Kennicott Glacier (41.4% of area) and 95.2% of total mass on Root Glacier (38.9% of area), indicating that the area covered by the generated DEMs from this study capture nearly all of the present-day mass change. The minimal mass change in the accumulation areas align with previous work, which shows thinning in the accumulation area from 1994-2013 converges to zero across much of Alaska[2].

Supplementary Note 2: Previous studies on Kennicott and Root Glaciers

As prominent features of the Wrangell-St. Elias National Park and Preserve, Kennicott and Root Glaciers are both relatively well-visited and well-studied. Past research has either focused on specific glacier processes–including surface hydrology, debris cover, and ice cliff evolution[3,4], subglacial hydrology and subseasonal velocity[5-7], and glacial lake evolution[8-10]–or broader-scale thinning[11,12]. Das et al. (2014)[11] calculated elevation changes across the Wrangell Mountains using U.S. Geological Survey (USGS) topographic maps from 1957 and DEMs from the 2000s (a comparison of the 1957 USGS topographic map derived DEM with this study's 1957 DEM is shown in Fig. S5), showing an accelerated mass change in the Wrangell Mountains in recent years. Anderson et al. (2021b)[12] focused on the Kennicott Glacier terminus with a similar suite of DEMs but incorporated the effects of ice dynamics (i.e., flux divergence) and surface features (i.e., ice cliffs, supraglacial streams, and debris) on thinning. Correlations were found using surface features, ice dynamics, and glacier thinning showing that reduced flow drives thinning patterns on the Kennicott Glacier terminus. We build on the insights gained from these studies by producing an extended time series of high-resolution DEMs every ~20 years from 1938 to 2023 across the ablation area of both Kennicott and Root Glaciers, incorporating bed data and historical velocities, enabling us to highlight the spatial distribution, timing, and magnitude of thinning and dynamic changes on Kennicott and Root Glaciers.

Supplementary Note 3: Historical image processing settings

Image preprocessing was performed using the open-source software MicMac v1.0.beta14[13]. In particular, scanned film images were normalized to the same grid by manually identifying fiducial marks using the *SaisieAppuisInitQT* command and resampled to 25 microns with the *ReSampFid* command. Historical DEM and orthomosaic generation was carried out in the structure-from-motion photogrammetric software Agisoft Metashape v1.8.2 (Agisoft, 2022) (Methods).

Supplementary Note 4: Historical glacier-wide mass balance

To facilitate comparisons between modeled and observed glacier-wide mass balances, the modeled annual mass balance was aggregated to align with DEM difference dates (i.e., 1938-1957, 1957-1978, 1978-2004, and 2000-2019). Specific mass balance from historical DEMs was calculated by converting volume change to mass change (assuming a density of 900 kg m^{-3}) and dividing by glacier area. As historical DEMs only covered the ablation area, uncertainty was incorporated to account for unmeasured mass balance in the accumulation area based on differences in the 1957 USGS topographic map (which spans the entire glacier) and the 1957 DEM produced from this study, assuming the accumulation area extent did not change. For the modeled mass balance prior to 1957 where DEM coverage was limited to the terminus of Kennicott Glacier, observations showed greater mass balance than from 1957-1978. Thus, we assumed that the glacier-wide mass balance from 1938-1957 was greater than that from 1957-1978.

2 SUPPLEMENTARY TABLES

Supplementary Table 1: Overview of used and produced datasets in this study, and whether the data product is existing, produced, or used for the first time ('new') in this study

Product	Year (MM-DD)	Source	Data	Usage	Notes
DEM	1938	Washburn	Oblique stereo photographs	New	Post-processed to detrend artifacts in DEM generated from oblique imagery
DEM	1957 (07-29)	USAF	NADIR stereo photographs	Produced	
DEM	1978 (08-28)	NASA	NADIR stereo photographs	Produced	
DEM	2004 (05-04)	ASTER	Optical stereo satellite images	Existing	Post-processed to fill holes and adjust DEM to late summer
DEM	2012 (08-14 to 09-08)	IFSAR	SAR	Existing	The DEM is from sometime between 14 August and 8 September
DEM	2023 (08-01)	NPS	Optical stereo images	New	
Bed topography	2023	NPS	Ice penetrating radar	New	
Velocity	1960	This study	NADIR stereo photographs	Produced	Product derived from surface displacements from 1957-1962

Velocity	1990-2018	ITS LIVE	Various optical satellite images	Existing	
Outline	1957	This study	NADIR stereo photographs	Produced	
Outline	2023	This study	Optical stereo images	Produced	
Climate data	1940-2023	ECMWF	Climate reanalysis data	Existing	
Climate data	1940-2100	CMIP6	Climate model projections	Existing	Data from 12 GCMs and 4 SSPs

Supplementary Table 2: Kennicott and Root Glaciers annual elevation change and mass loss. Total mass loss (i.e., the sum of mass loss from the Kennicott and Root Glaciers ablation areas) represents ~95% of glacier-wide mass loss.

Time Span		Kennicott Terminus		Kennicott		Root		Total	
Start	End	[m yr^{-1}]	[Mt yr^{-1}]	[m yr^{-1}]	[Mt yr^{-1}]	[m yr^{-1}]	[Mt yr^{-1}]	[m yr^{-1}]	[Mt yr^{-1}]
1938	1957	0.621 ± 0.924	9.95 ± 14.80	--	--	--	--	--	--
1957	1978	-0.332 ± 0.142	-5.31 ± 2.28	-0.441 ± 0.018	-45.94 ± 2.57	-0.245 ± 0.015	-7.50 ± 0.43	-0.397 ± 0.018	-53.44 ± 2.97
1978	2004	-1.843 ± 0.259	-29.53 ± 4.08	-0.736 ± 0.031	-76.67 ± 4.33	-0.846 ± 0.054	-25.89 ± 1.56	-0.761 ± 0.035	-102.57 ± 5.79
2004	2012	-2.097 ± 0.975	-28.65 ± 12.50	-1.548 ± 0.070	-155.93 ± 9.07	-1.493 ± 0.108	-44.55 ± 3.06	-1.532 ± 0.077	-200.48 ± 11.81
2012	2023	-1.977 ± 0.201	-22.42 ± 2.04	-1.430 ± 0.061	-139.09 ± 7.75	-1.410 ± 0.089	-40.97 ± 2.33	-1.418 ± 0.066	-180.08 ± 10.01
1957 Area [km^2]		17.8 ± 1.1		115.7 ± 4.7		34.0 ± 2.0		149.7 ± 6.7	
2023 Area [km^2]		12.6 ± 0.9		108.1 ± 4.6		32.3 ± 2.0		141.1 ± 6.6	

Supplementary Table 3: Historical image processing primary statistics.

Year	Images used	Ground control points	Ground control point error [m]	Dense cloud points [10^6]	DEM grid resolution [m]
1938	3	9	79.8	15.4	10
1957	32	77	3.21	47.3	4.5
1962	5	30	0.81	7.6	5.3
1978	17	81	1.04	12.6	12.6

Supplementary Table 4: Radar-derived ice thickness crossover disagreement.*

Crossings	Count	Mean	Std.	25%	50%	75%
AirIPR-AirIPR	278	14	22	2	6	18
AirIPR-Groundhog	18	23	21	10	20	28
Total	296	14	22	2	6	19

*Note: all units are in meters (except for count)

3 SUPPLEMENTARY FIGURES

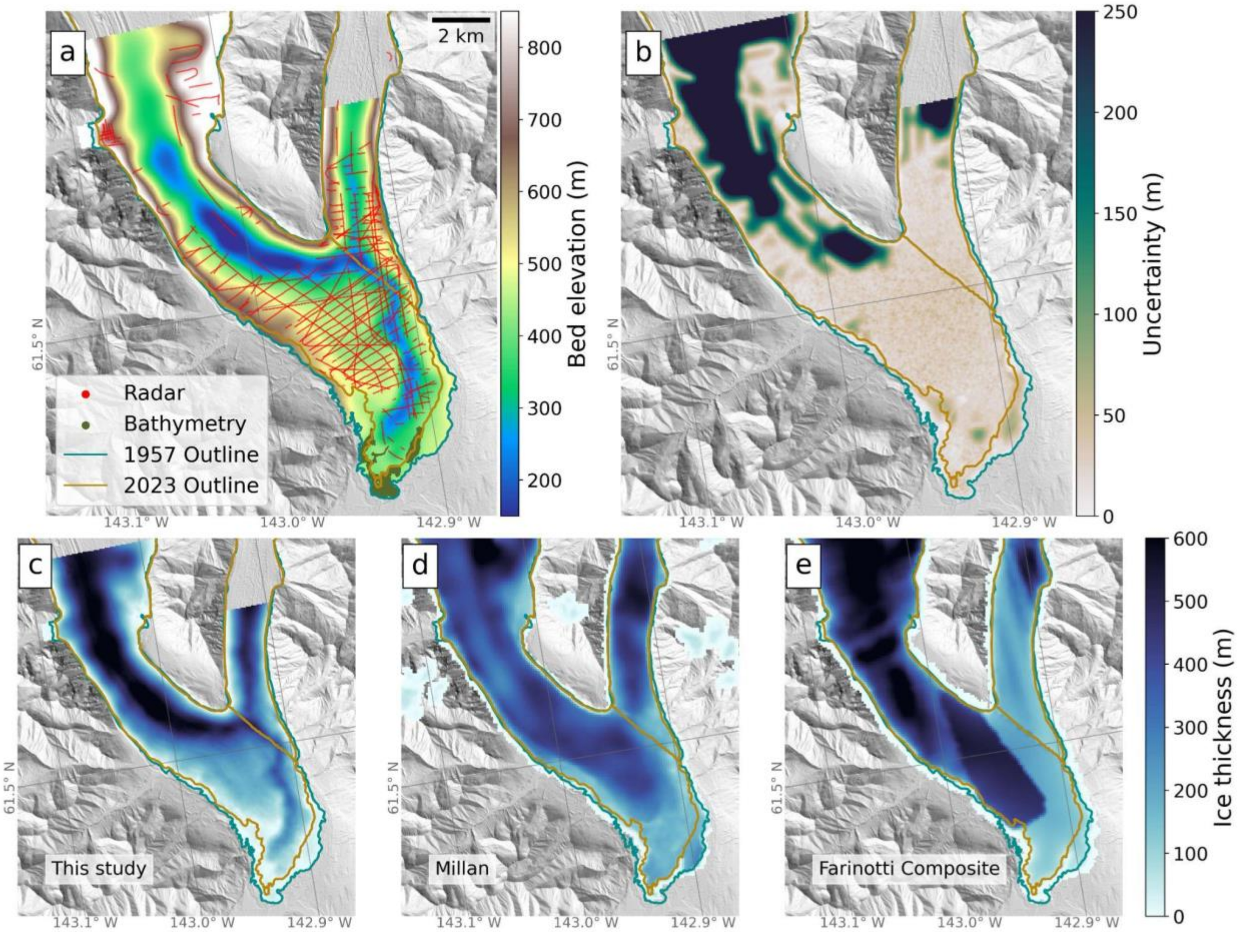

Supplementary Fig. 1: Kennicott and Root Glaciers ice-penetrating radar and proglacial lake bathymetry data points with the resulting interpolated bed elevation (a) and 2-sigma bed elevation uncertainty (b). A comparison of ice thickness between the ice-penetrating radar dataset (c), Millan et al. (2022)[14] (d), and the Farinotti et al. (2019)[15] composite (e) are shown. To facilitate comparison with other datasets (d and e) the ice thickness from this study, derived using the 2012 IFSAR DEM, is corrected with elevation change from Hugonnet et al. (2021)[1] to adjust (c) to the year 2000 to match (d) and (e).

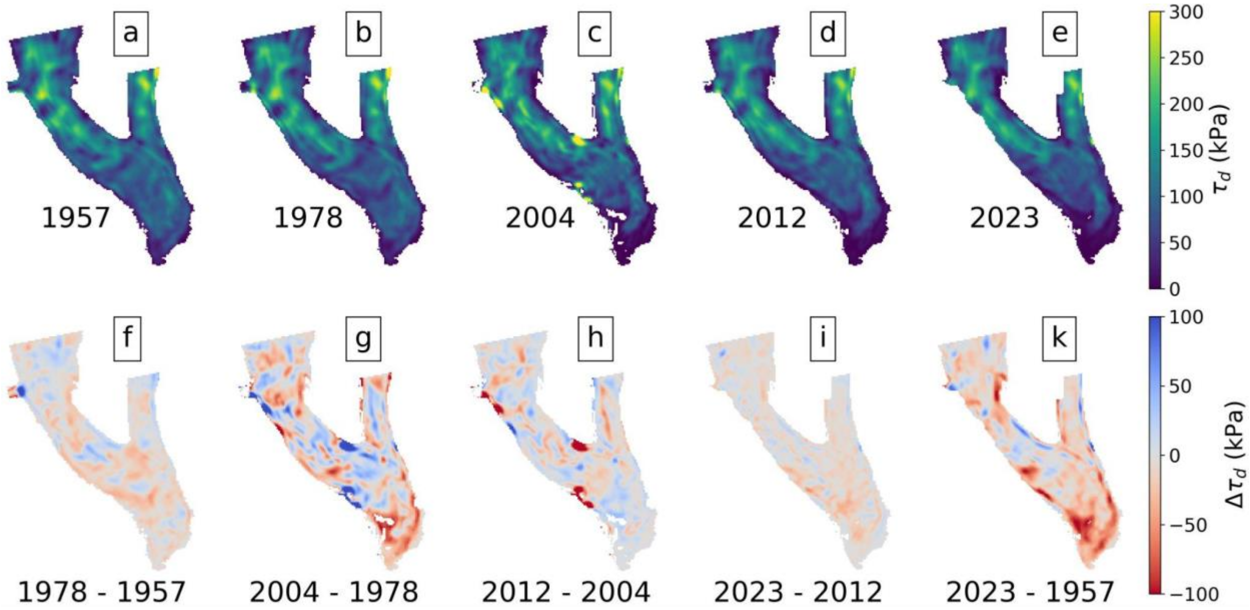

Supplementary Fig. 2: Kennicott and Root Glaciers spatially distributed driving stress (a-e) and change in driving stress (f-k). The 1957 glacier outline is used (a-k) to show changes over all ice-covered parts of the terrain since 1957.

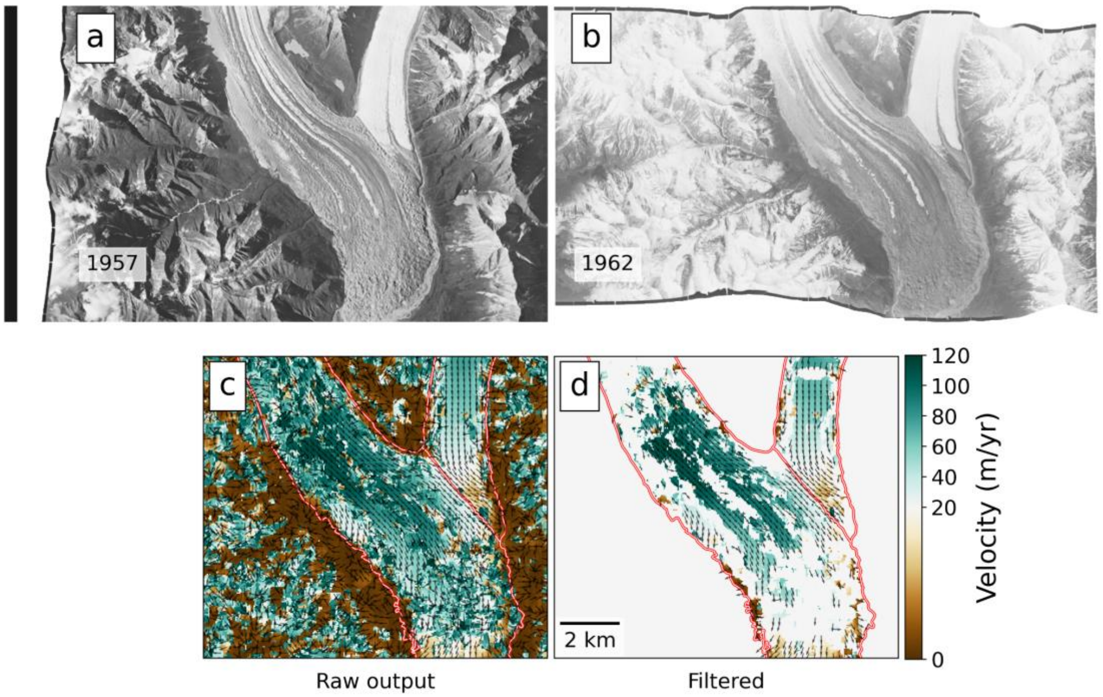

Supplementary Fig. 3: Velocity processing from historical image feature-tracking with PyCorr. Orthorectified film positives from 1957 (a) and 1962 (b) are shown with the resulting raw (c) and filtered (d) data outputs. Arrows represent direction and color indicates velocity magnitude.

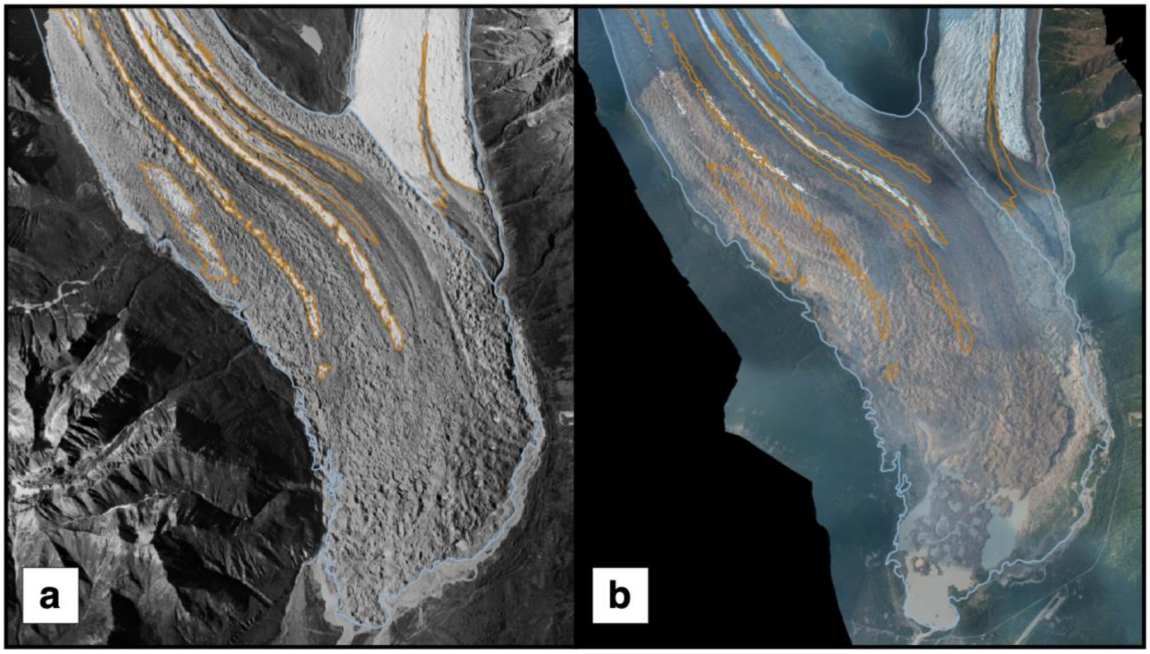

Supplementary Fig. 4: Kennicott and Root Glaciers orthophotos from (a) 1957 and (b) 2023. The 1957 outline (light blue) and approximate 1957 clean-ice extent (orange) are shown in both (a) and (b).

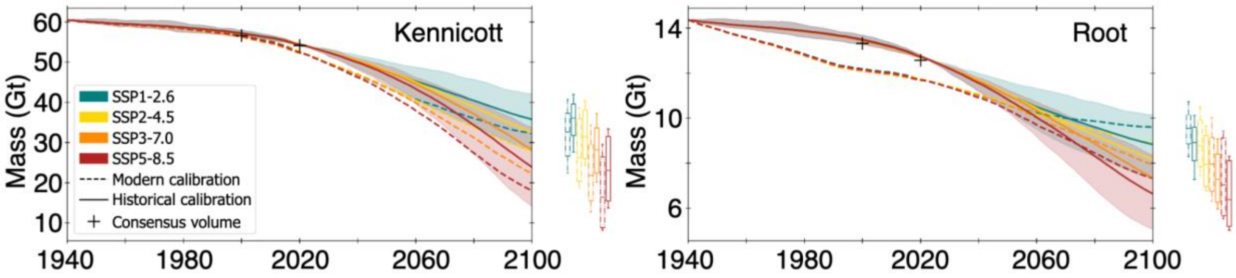

Supplementary Fig. 5: Kennicott and Root Glaciers mass from 1940-2100 for different shared socioeconomic pathways (SSPs). The mean of 12 GCM realizations for each SSP is plotted with a thick line. Shaded regions represent the minimum and maximum realization for an SSP scenario, and the box-plots show the distribution for each SSP in 2100. Box-plot edges represent data quartiles and the whiskers show the most extreme point 1.5 times the interquartile range. Results are shown using the optimal parameter set using historical data and modern data.

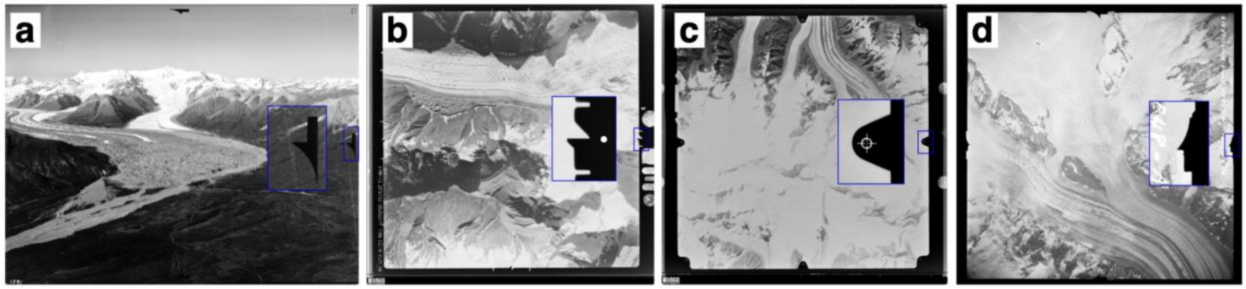

Supplementary Fig. 6: Examples of fiducial marks used for historical imagery internal orientation. The white dot in (b) and the crosshairs in (c) are exaggerated to make it clearly visible. These images are courtesy of the (a) Bradford Washburn Collection at the University of Alaska Fairbanks and (b-c) the U.S. Geological Survey.

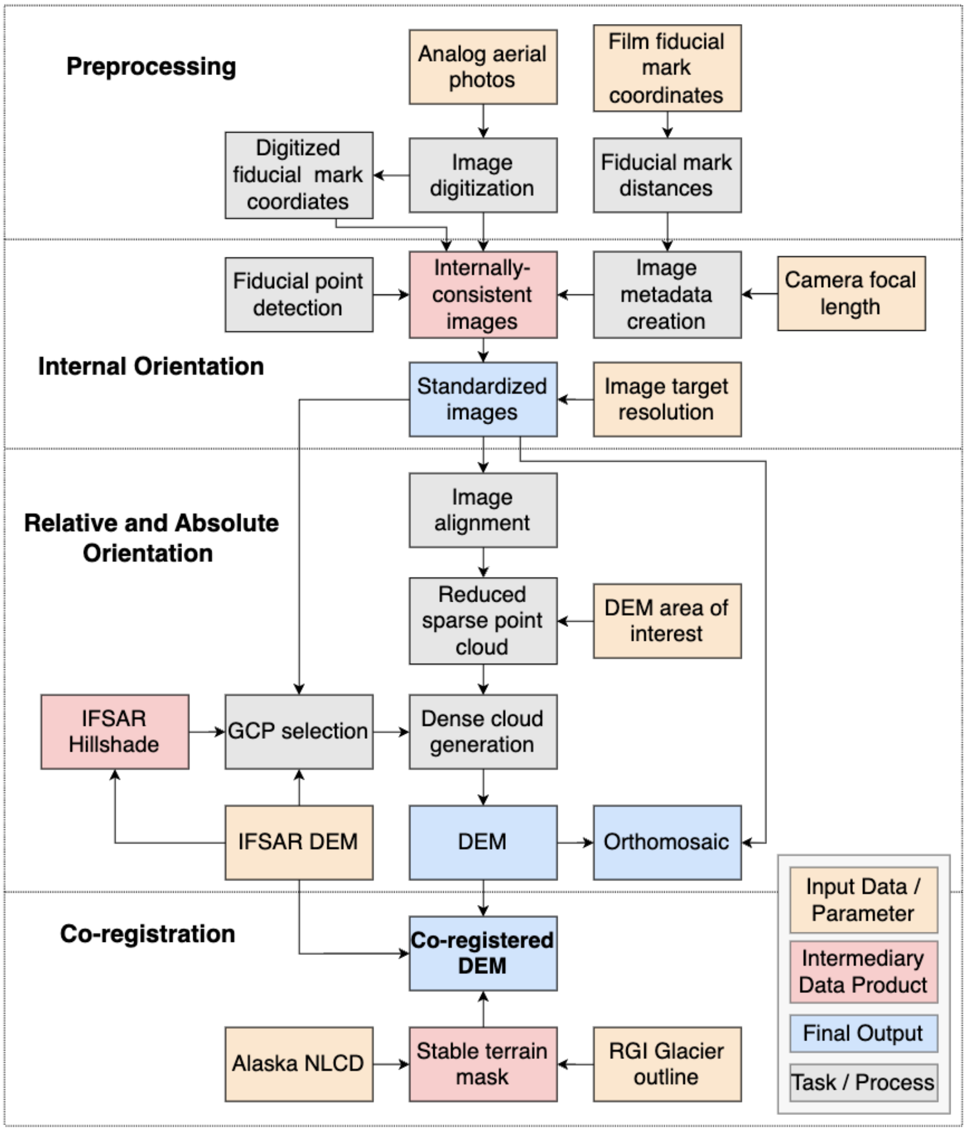

Supplementary Fig. 7: Schematic outlining historical DEM processing workflow.

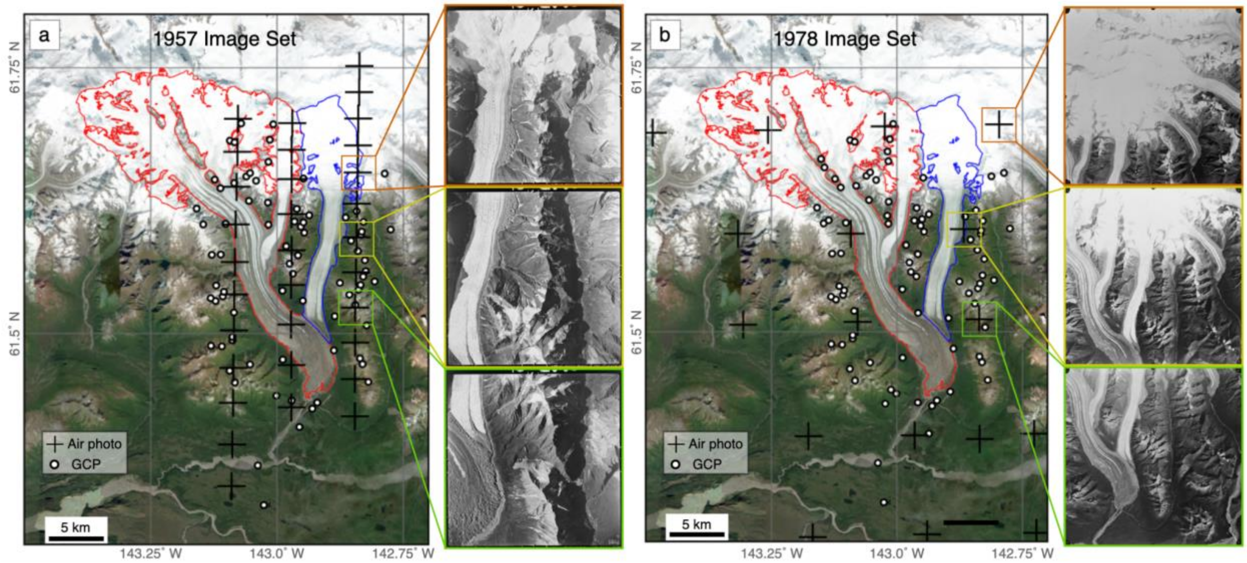

Supplementary Fig. 8: Aerial photograph and ground-control point (GCP) locations for the 1957 (a) and 1978 (b) data. Examples of individual air photos are shown to the right of each overview schematic. The background map in (a) and (b) is the intellectual property of Esri and is used herein under license. Copyright © 2025 Esri and its licensors, all rights reserved.

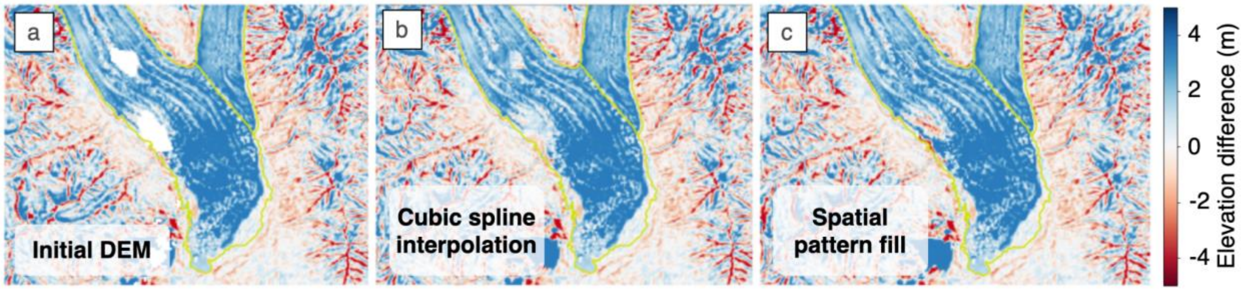

Supplementary Fig. 9: Results from the various hole-filling techniques applied to the ASTER DEM. The initial DEM product from 4 May 2004 (a) filled with a cubic spline interpolation (b) or with long-term thinning pattern (c). Hole-filling is conducted on the DEM difference, relative to the 2012 IFSAR DEM[16].

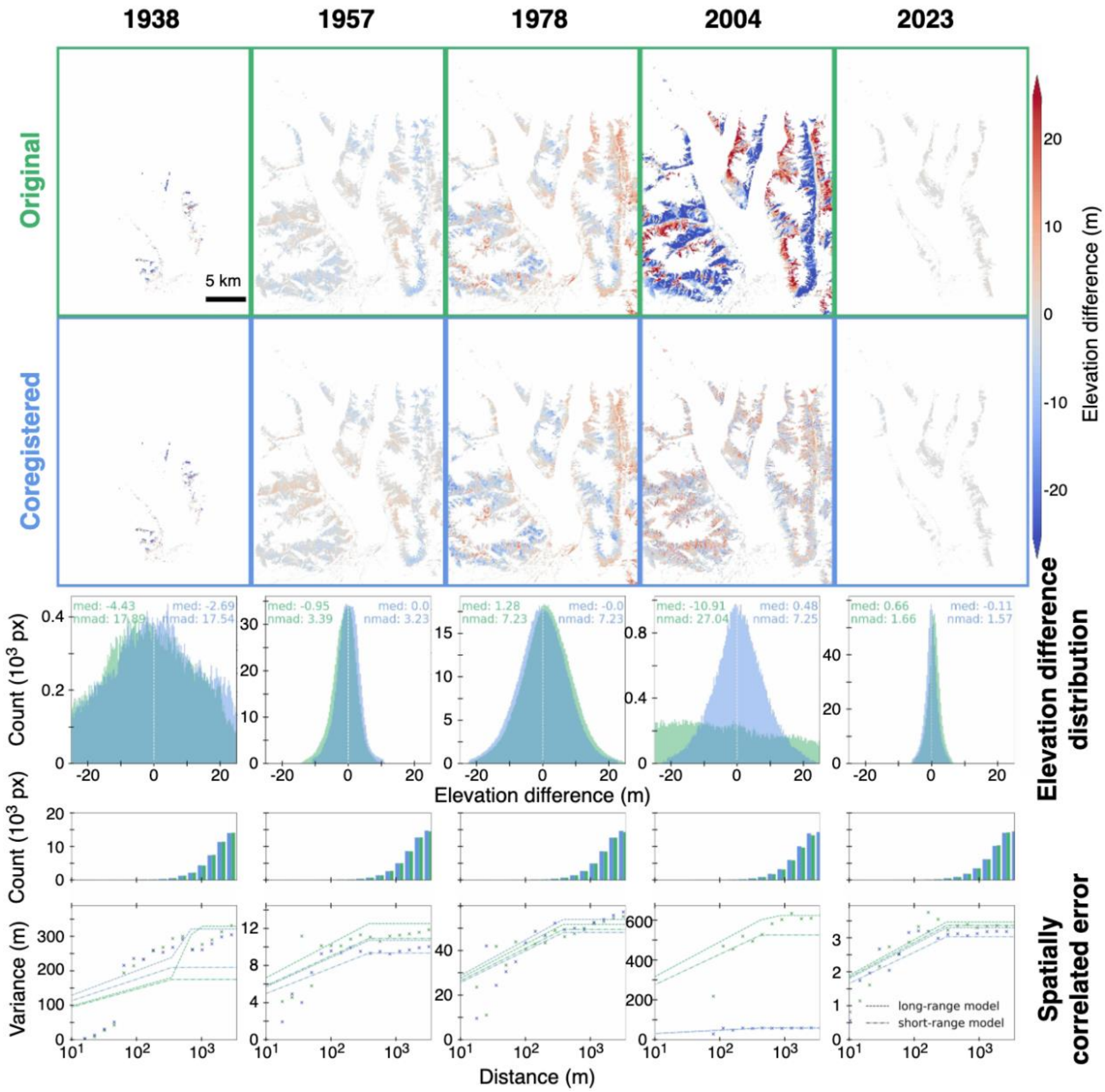

Supplementary Fig. 10: DEM co-registration accuracy and results. Elevation difference (from IFSAR) of stable terrain before (green) and after (blue) co-registration (top panels) with corresponding histogram of stable terrain elevation difference (middle) is shown for each DEM. Residual distribution and empirical variogram with short- and long-range model (bottom) is similarly shown.

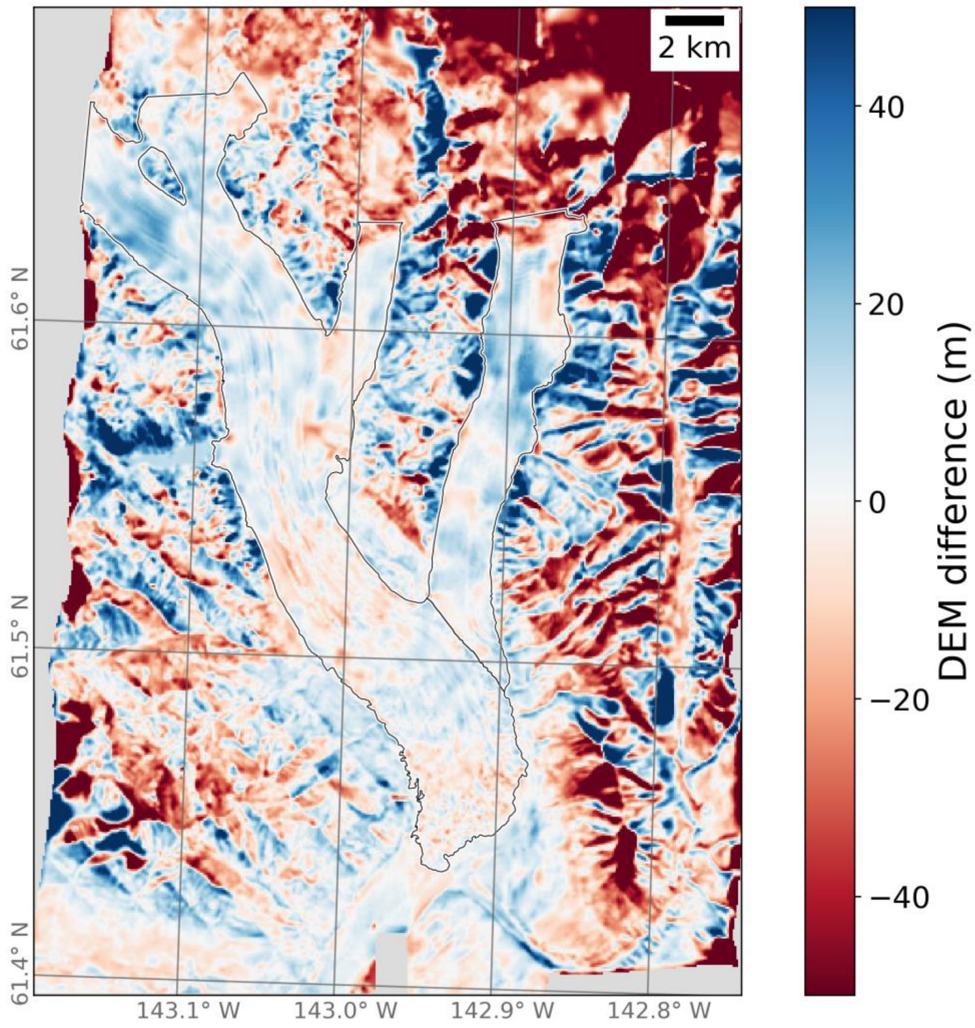

Supplementary Fig. 11: Map showing elevation differences between the 1957 USGS topographic DEM and the 1957 DEM produced in this study. Mean difference of glaciated terrain in the ablation area is 3.57 m (3.09 m on Kennicott Glacier and 5.18 m on Root Glacier). Positive values indicate higher elevation in the 1957 DEM from this study compared to the USGS topographic DEM product.

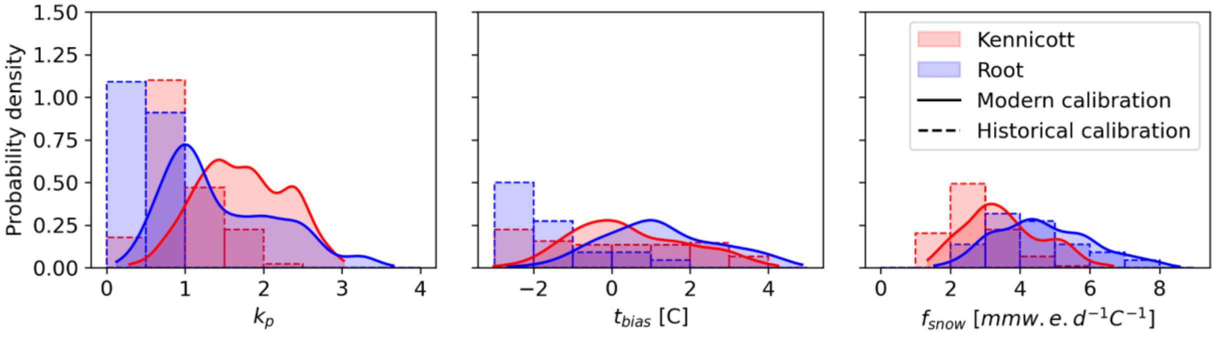

Supplementary Fig. 12: PyGEM precipitation factor (left), temperature bias (center), and degree-day factor of snow (right) distributions for historical and modern calibrations. Modern calibration follows a Bayesian framework to obtain probabilistic distributions of each parameter[17]. Historical calibration takes parameter sets that align with long-term mass balance records (n=89 for Kennicott Glacier, n=22 for Root Glacier), where histograms of the individual parameter distributions are shown.

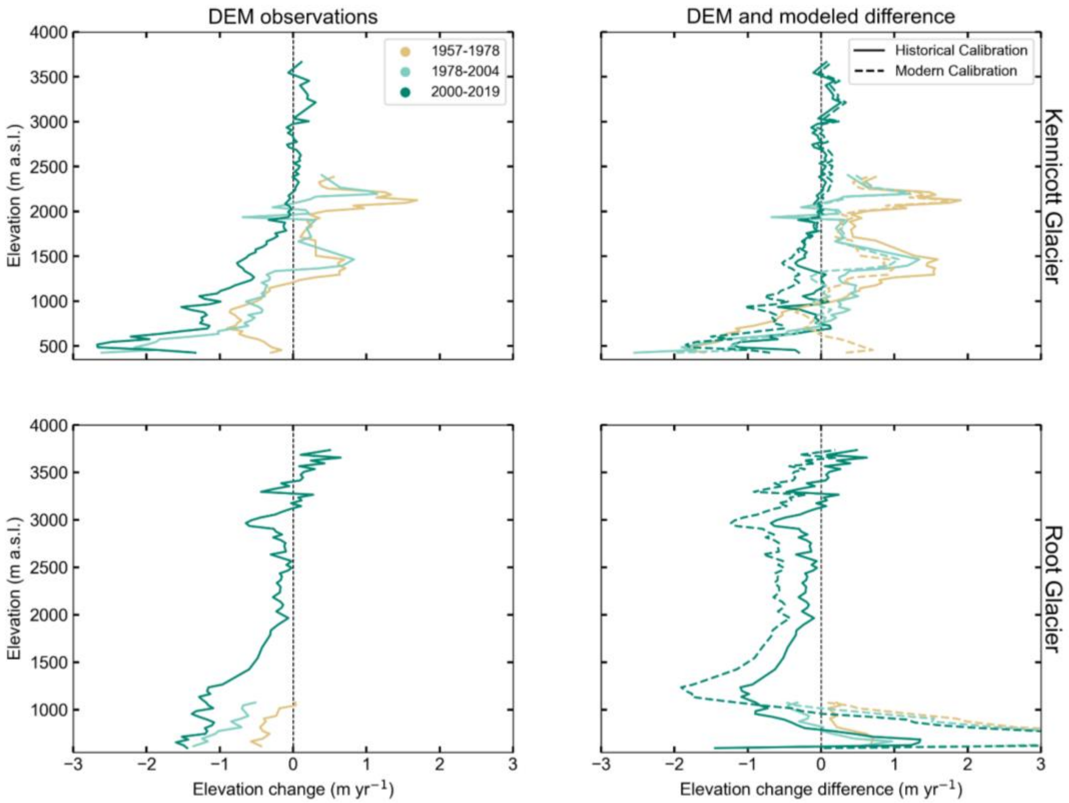

Supplementary Fig. 13: Elevation-binned surface elevation change from DEM observations (left) and elevation change difference between geodetic and modeled estimates (right) for Kennicott (top) and Root (bottom) Glaciers. Results are shown from 1957-1978, 1978-2004, and 2000-2019. Model calibration with historical data shows less bias on elevation change rates compared to observations, including from 2000-2019.

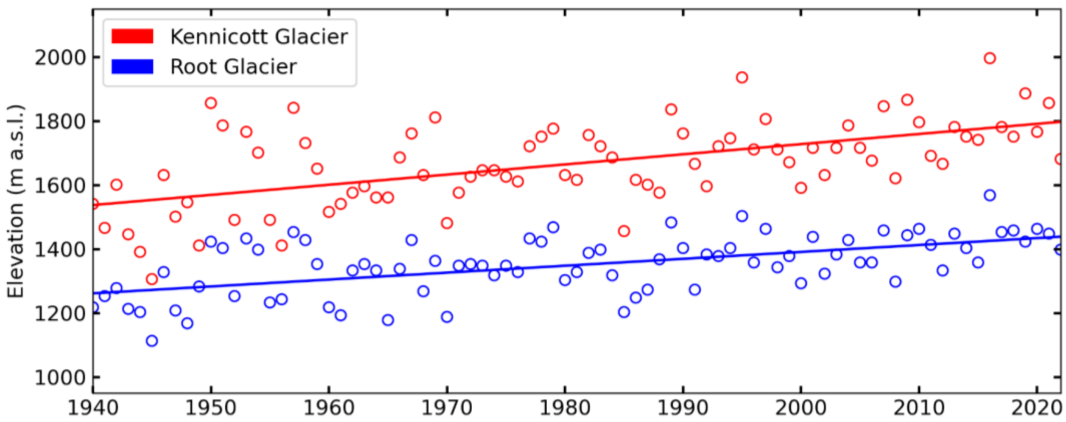

Supplementary Fig. 14: Kennicott and Root Glacier equilibrium-line altitude from 1940-2022 for the historically-calibrated model. The points represent the equilibrium-line altitude for individual years with a line of best fit showing long-term trends.

4 SUPPLEMENTARY MATERIAL REFERENCES

1. Hugonnet, R. *et al.* Accelerated global glacier mass loss in the early twenty-first century. *Nature* **592**, 726–731 (2021).
2. Larsen, C. F. *et al.* Surface melt dominates Alaska glacier mass balance. *Geophysical Research Letters* **42**, 5902–5908 (2015).
3. Anderson, L. S., Armstrong, W. H., Anderson, R. S. & Buri, P. Debris cover and the thinning of Kennicott Glacier, Alaska: in situ measurements, automated ice cliff delineation and distributed melt estimates. *The Cryosphere* **15**, 265–282 (2021a).
4. Petersen, E., Hock, R. & Loso, M. G. Stream hydrology controls on ice cliff evolution and survival on debris-covered glaciers. *Earth Surface Dynamics* **12**, 727–745 (2024).
5. Bartholomaus, T. C., Anderson, R. S. & Anderson, S. P. Response of glacier basal motion to transient water storage. *Nature Geosci* **1**, 33–37 (2008).
6. Bartholomaus, T. C., Anderson, R. S. & Anderson, S. P. Growth and collapse of the distributed subglacial hydrologic system of Kennicott Glacier, Alaska, USA, and its effects on basal motion. *J. Glaciol.* **57**, 985–1002 (2011).
7. Armstrong, W. H., Anderson, R. S., Allen, J. & Rajaram, H. Modeling the WorldView-derived seasonal velocity evolution of Kennicott Glacier, Alaska. *Journal of Glaciology* **62**, 763–777 (2016).
8. Anderson, S. P. *et al.* Integrated hydrologic and hydrochemical observations of Hidden Creek Lake jökulhlaups, Kennicott Glacier, Alaska. *Journal of Geophysical Research: Earth Surface* **108**, (2003).
9. Anderson, S. P., Longacre, S. A. & Kraal, E. R. Patterns of water chemistry and discharge in the glacier-fed Kennicott River, Alaska: evidence for subglacial water storage cycles. *Chemical Geology* **202**, 297–312 (2003).

10. Armstrong, W. H. & Anderson, R. S. Ice-marginal lake hydrology and the seasonal dynamical evolution of Kennicott Glacier, Alaska. *Journal of Glaciology* **66**, 699–713 (2020).
11. Das, I., Hock, R., Berthier, E. & Lingle, C. S. 21st-century increase in glacier mass loss in the Wrangell Mountains, Alaska, USA, from airborne laser altimetry and satellite stereo imagery. *Journal of Glaciology* **60**, 283–293 (2014).
12. Anderson, L. S., Armstrong, W. H., Anderson, R. S., Scherler, D. & Petersen, E. The Causes of Debris-Covered Glacier Thinning: Evidence for the Importance of Ice Dynamics From Kennicott Glacier, Alaska. *Frontiers in Earth Science* **9**, (2021b).
13. Rupnik, E., Daakir, M. & Pierrot Deseilligny, M. MicMac – a free, open-source solution for photogrammetry. *Open geospatial data, softw. stand.* **2**, 14 (2017).
14. Millan, R., Mouginot, J., Rabatel, A. & Morlighem, M. Ice velocity and thickness of the world's glaciers. *Nat. Geosci.* **15**, 124–129 (2022).
15. Farinotti, D. *et al.* A consensus estimate for the ice thickness distribution of all glaciers on Earth. *Nat. Geosci.* **12**, 168–173 (2019).
16. McNabb, R., Nuth, C., Kääb, A. & Girod, L. Sensitivity of glacier volume change estimation to DEM void interpolation. *The Cryosphere* **13**, 895–910 (2019).
17. Rounce, D. R. *et al.* Global glacier change in the 21st century: Every increase in temperature matters. *Science* **379**, 78–83 (2023).
